# PPARα Is Essential for Microparticle-Induced Differentiation of Mouse Bone Marrow-Derived Endothelial Progenitor Cells and Angiogenesis

**DOI:** 10.1371/journal.pone.0012392

**Published:** 2010-08-25

**Authors:** Tarek Benameur, Simon Tual-Chalot, Ramaroson Andriantsitohaina, María Carmen Martínez

**Affiliations:** 1 CNRS, UMR 6214, Angers, France; 2 INSERM, U771, Angers, France; 3 Faculté de Médecine, Université d'Angers, Angers, France; University of Tor Vergata, Italy

## Abstract

**Background:**

Bone marrow-derived endothelial progenitor cells (EPCs) are critical for neovascularization. We hypothesized that microparticles (MPs), small fragments generated from the plasma membrane, can activate angiogenic programming of EPCs.

**Methodology/Principal Findings:**

We studied the effects of MPs obtained from wild type (MPs^PPARα+/+^) and knock-out (MPs^PPARα−/−^) mice on EPC differentiation and angiogenesis. Bone marrow-derived cells were isolated from WT or KO mice and were cultured in the presence of MPs^PPARα+/+^ or MPs^PPARα−/−^ obtained from blood of mice. Only MPs^PPARα+/+^ harboring PPAR^α^ significantly increased EPC, but not monocytic, differentiation. Bone marrow-derived cells treated with MPs^PPARα+/+^ displayed increased expression of pro-angiogenic genes and increased *in vivo* angiogenesis. MPs^PPARα+/+^ increased capillary-like tube formation of endothelial cells that was associated with enhanced expressions of endothelial cell-specific markers. Finally, the effects of MPs^PPARα+/+^ were mediated by NF-κB-dependent mechanisms.

**Conclusions/Significance:**

Our results underscore the obligatory role of PPARα carried by MPs for EPC differentiation and angiogenesis. PPARα-NF-κB-Akt pathways may play a pivotal stimulatory role for neovascularization, which may, at least in part, be mediated by bone marrow-derived EPCs. Improvement of EPC differentiation may represent a useful strategy during reparative neovascularization.

## Introduction

Bone marrow-derived endothelial progenitor cells (EPCs) possess the capacity to proliferate, migrate, and differentiate into endothelial lineage cells [1]. EPCs are mobilized from bone marrow into the circulation during vascular injury, and then home to the site of neovascularization and thereby contribute to postnatal neovascularization [2]–[4]. Moreover, decreased levels of EPCs correlate with subsequent increases in cardiovascular events suggesting that EPCs are important predictors for cardiovascular mortality and morbidity [5], [6]. It has also been proposed that other populations of bone marrow-derived cells that are phenotypically different from EPCs can also be recruited to the injured endothelium and contribute to the regeneration of the endothelium [7]. Characterization of these cells indicates that they mainly express hematopoietic lineage markers such as CD11b, CD14 and CD45 [8], whereas EPCs express CD31, CD106, and Flk-1 [9].

One potential limitation for the use of autologous bone marrow EPCs for therapeutic vasculogenesis is the decline in the number and function of progenitor cells in patients with coronary artery disease [10], diabetes [11], metabolic syndrome [12] and severe heart failure [13]. Therefore, enhancing EPC differentiation and homing towards sites of injury will likely improve efficiency of cell therapy; this approach is a subject of intense investigation. Emerging evidence indicates that PPARα, a member of the nuclear hormone receptor superfamily activated by ligands and several fatty acids [14], improves nitric oxide (NO)-mediated vasodilatation in conductance and resistance vessels and also protects against myocardial ischemic injury [15].Recent reports suggest that stimulation of PPARα, but not PPARβ or PPARγ, enhances cardiac progenitor differentiation through the involvement of NADPH oxidase activity [16], [17] and that activation of PPARα induces neovascularization through a VEGF-dependent mechanism [18], [19]. Collectively these studies suggest that stimulation of PPARα may stimulate cell differentiation.

The role of cell-to-cell communication involving membrane fragments such as microparticles (MPs) [20], [21] during neovascularization is unknown. MPs are small vesicles released from the plasma membrane of stimulated or apoptotic cells; their protein composition is not completely elucidated, but it differs in function depending on the cell origin and the stimuli for their generation [22]. Importantly, MPs can transfer receptors and organelles between cells and deliver mRNA and proteins into cells [20]. Thus, MPs can be considered as real vectors of biological messages such as induction of angiogenesis or differentiation since endothelial, platelet or lymphocytic MPs can modulate *in vitro* and *in vivo* angiogenic properties of EPCs and ECs [23]–[26]. However, the mechanism(s) by which circulating MPs modulate angiogenesis have not elucidated. Because PPARs can be harbored by MPs [27], we hypothesized that the effects of circulating MPs on EPC differentiation may be mediated, at least in part, by the PPARα pathway. Therefore, the aim of this study was to determine whether circulating MPs induce bone marrow-derived cell differentiation towards an endothelial lineage and increase angiogenic-related properties of these cells and ECs.

## Results

### The number and origin of circulating MPs does not differ between wild type (WT) and PPARα null mice

**Figure 1:**
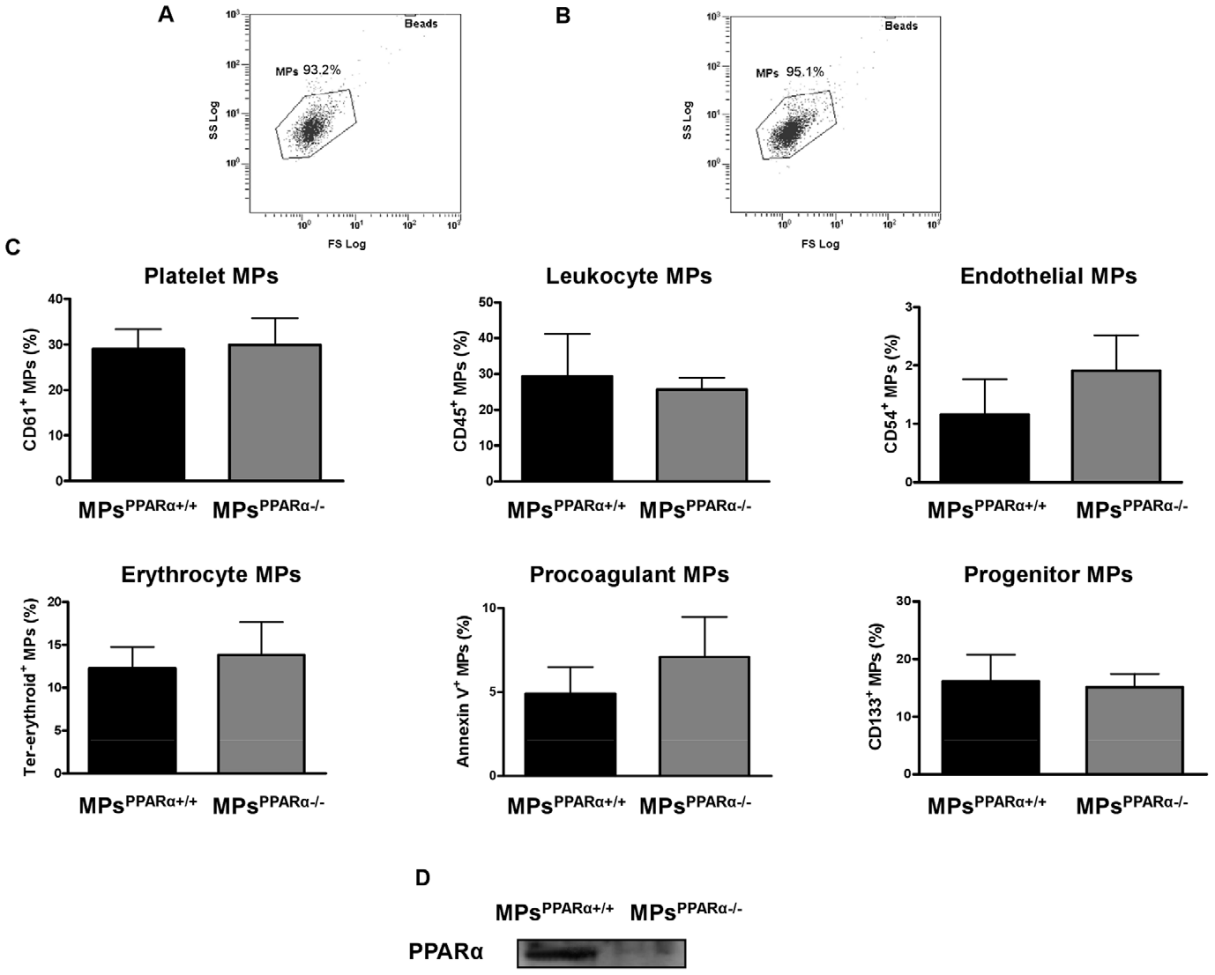
Phenotypic characterization of circulating MP levels in PPARα+/+ mice compared to PPARα−/− mice. Circulating MPs from PPARα+/+ (A) and PPARα−/− (B) mice and Flowcount beads (Beads region, 10 µm diameter) are visualized in a side scatter (SS)/forward scatter (FS) logarithmic representation. MPs are defined as events with size 0.1 to 1 µm gated in the “MPs” window. (**C**) Different populations of MPs derived from platelets, leukocytes, endothelial cells, erythrocytes, procoagulant MPs and MPs from progenitor cells. There was no significant difference in circulating MP origins between PPARα−/− and their WT littermate mice. (**D**) Western blot indicates that MPs isolated from PPARα WT mice blood harboured PPARα, while those isolated from PPARα−/− mice blood did not. Figures are representative of at least 5 independent experiments for each condition.

Flow cytometry analysis of circulating MPs (Figure 1A and B) showed that the total number of circulating MPs was not significantly different between PPARα null mice (2056±360 MPs/µL plasma) and their corresponding WT littermates (1678±417 MPs/µL plasma), indicating that PPARα deletion did not affect the circulating levels of MPs (Figure 1C). These MP concentrations were used for further experiments. There was also no significant differences in the cellular origins of MPs between the two groups of mice (Figure 1C) with only MPs from PPARα WT (MPs^PPARα+/+^) expressing PPARα receptors (Figure 1D).

### Circulating MPs^PPARα+/+^, but not MPs^PPARα−/−^, increase the differentiation of bone marrow-derived cells towards the EPC lineage

Phenotypic characterization of bone marrow-derived cells using multiple markers indicates that, at day 7, the presence of populations of bone marrow-derived cells displaying double labeling of Sca-1^+^/CD31^+^, Sca-1^+^/CD45^+^, Sca-1^+^/CD14^+^ and F4/80^+^/CD31^+^ whose levels were enhanced compared to those at day 0 (Figure S1). It should be noted that the percentages of bone marrow-derived cells expressing Sca-1^+^/Flk-1^+^ and CD133^+^/Flk-1^+^ were not significantly different at day 0 and 7. This characterization indicates that, at day 7, bone marrow-derived cells are mainly cells expressing monocytic and endothelial markers; and moreover that the EPC subpopulation belongs to the so-called “early EPCs”.

**Figure 2:**
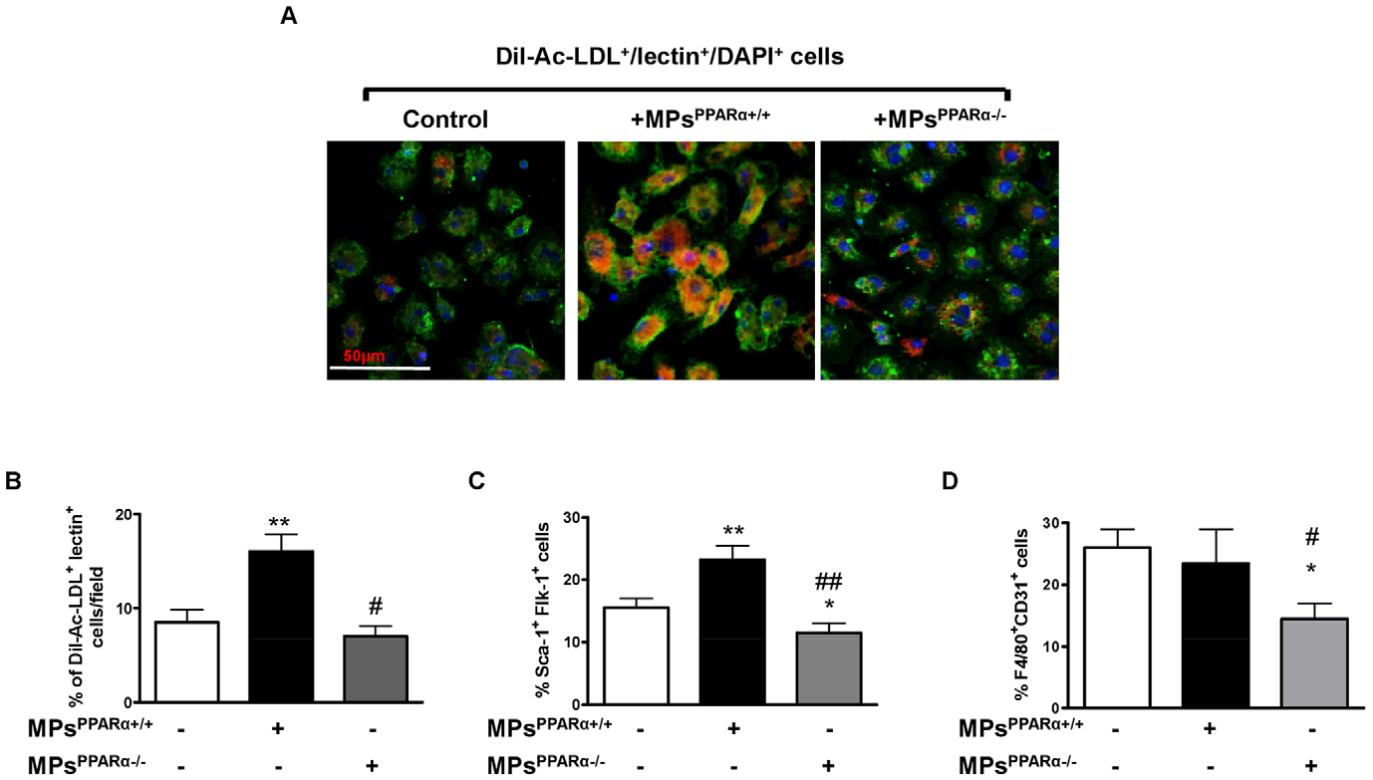
MPs^PPARα+/+^ treatment increases the number of EPC undergoing *in vitro* differentiation. (**A**) Confocal microscopy characterization of mice bone marrow-derived EPC differentiation: cells were incubated in the absence or in the presence of either MPs^PPARα+/+^ or MPs^PPARα−/−^. After 7 days in culture, cells with endothelial phenotype were characterized as adherent cells with double-positive staining for Dil-acetylated-LDL (red) and lectin (green). Nuclei were counterstained with DAPI (blue). (**B**) Quantification of Dil-acetylated-LDL- and lectin-positive cells derived from bone marrow in the absence or in the presence of either MPs^PPARα+/+^ or MPs^PPARα−/−^. Histogram showing the effects of MPs either from PPARα+/+ or PPARα−/− mice, on the differentiation of EPCs. (**C**) Flow cytometric characterization of EPC differentiation. Cells with double-positive staining for Sca-1^+^/Flk-1^+^ were identified as differentiating EPCs. (**D**) Flow cytometric characterization of macrophage differentiation. Cells with double-positive staining for F4/80^+^/CD311^+^ were identified as macrophages. Data are shown as mean values±SEM of at least 3 independent experiments for each condition. *P<0.05, **P<0.01, ***P<0.001 *vs* in the absence of MPs (control); #*P*<0.05, ##*P*<0.01 *vs* in the presence of MPs^PPARα+/+^.

To investigate the effects of PPARα carried by MPs on *in vitro* bone marrow-derived cell differentiation, circulating MPs isolated from PPARα-deficient or WT mice were added to adherent bone marrow-derived cells from WT mice. Thereafter, EPCs were identified by Dil-Acetyl-LDL-uptake with concomitant lectin binding, as previously described [28], and double expression of Sca-1 and Flk-1 determined. After 7 days, isolated bone marrow-derived cells cultured in the presence of MPs^PPARα+/+^ displayed enhanced adhesion but with no changes in cell proliferation (data not shown). At day 7, confocal microscopy characterization of adherent cells incubated with MPs^PPARα+/+^, but not with MPs^PPARα−/−^, also demonstrated increased Dil-ac-LDL/lectin uptake typical for endothelial lineage (Figure 2A). As illustrated in Figure 2B, quantification indicates that only MPs^PPARα+/+^ significantly increased the number of double-positive (Dil-Ac-LDL^+^/lectin^+^) cells. Similar results were obtained by flow cytometry, revealing double-positive staining with Sca-1/Flk-1. Indeed, MPs^PPARα+/+^ increased the number of differentiated EPC-like attaching cells by 36% (Figure 2C). In contrast, the number of Sca-1^+^Flk-1^+^ cells were significantly lower in EPCs treated with MPs^PPARα−/−^ when compared with non-treated EPCs (Figure 2C). In addition, MPs^PPARα+/+^ had no effect on the number of cells expressing both F4/80 and CD31 markers whereas the number of F4/80^+^CD31^+^ cells treated with MPs^PPARα−/−^ were significantly reduced (Figure 2D).

### Pharmacological PPARα activation induces EPC differentiation

To evaluate the presence of a functional PPARα pathway in bone marrow-derived cells and also whether direct activation of PPARα leads to EPC differentiation, we first analyzed PPARα expression in bone marrow-derived cells and secondly, we used a selective PPARα agonist, WY-14643 (5 µM) to stimulate EPC differentiation of cells isolated from WT mice. As shown by Western blot, PPARα was expressed on bone marrow-derived cells at day 7 of culture (Figure S2A) and that PPARα levels were not modified in bone marrow-derived cells after incubation with MPs^PPARα+/+^ (Figure S2A), suggesting that PPARα transfer between MPs and bone marrow-derived cells did not take place. Interestingly, the PPARα agonist significantly increased (∼42%) EPC differentiation from WT but not from PPARα-null mice as assessed by Sca-1^+^/Flk-1^+^ staining (Figure S2B and S2C). In addition, when bone marrow-derived cells from PPARα-null mice were treated with MPs from WT mice, no changes in EPC differentiation were observed (Figure S2D). Altogether, these results demonstrated the presence of functional PPARα receptors on bone marrow-derived cells from WT mice that induce differentiation and that it was unlikely that PPARα protein was transferred from MPs to bone marrow-derived cells.

### MPs^PPARα+/+^-induced EPC differentiation is associated with up-regulation of pro-angiogenic factor mRNA levels

**Figure 3:**
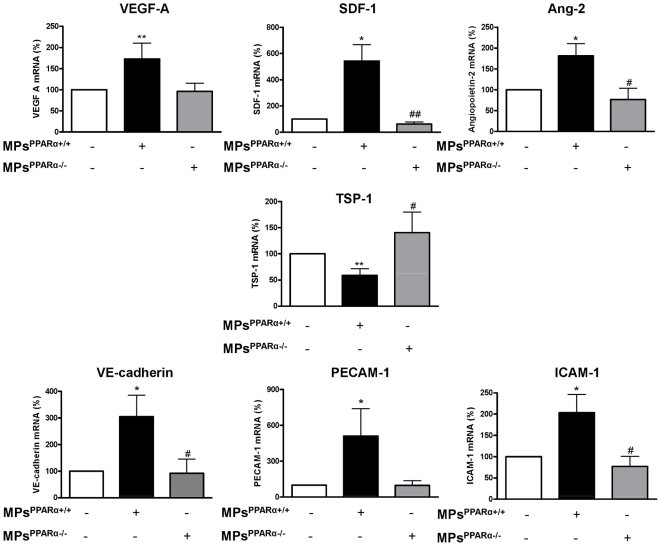
MPs^PPARα+/+^ upregulate mRNA levels of pro-angiogenic factors and SDF-1 in differentiating bone marrow-derived cells. Quantitative RT-PCR analysis of pro-angiogenic factors in cultured EPCs demonstrated that MPs^PPARα+/+^, but not MPs^PPARα−/−^, increased mRNA levels of VE-cadherin, ICAM-1, Ang-2, VEGF-A, PECAM-1 and SDF-1. TSP-1 mRNA level was down-regulated in MPs^PPARα+/+^-treated EPCs whereas up-regulated in MPs^PPARα−/−^-treated EPCs. *P<0.05, **P<0.01 *vs* in the absence of MPs; #*P*<0.05, ##*P*<0.01 *vs* in the presence of MPs^PPARα+/+^. Figures are representative of at least 3 independent experiments for each condition.

To confirm and extend the results obtained by immunofluorescence and flow cytometry, we further analyzed the expression of differentiation marker genes profiles by q-PCR. Mice endothelial markers and angiogenic gene expression were analyzed using a panel of 52 transcripts (Table S1). The data in Figure 3, which was obtained from cultured bone marrow-derived cells, shows that MPs^PPARα+/+^ enhances the expression of pro-angiogenic factors such as VEGF A, SDF-1, and angiopoietin-2, while the expression of thrombospondin-1 (TSP-1), which has a potent anti-angiogenic activity, was significantly reduced. In addition, mRNA levels of endothelial markers such as VE-cadherin, PECAM-1 and ICAM-1 were increased. Any significant changes of mRNA levels of pro-angiogenic factors and endothelial markers were observed when bone marrow-derived cells were treated with MPs^PPARα−/−^.

### MPs^PPARα+/+^ enhance EC-specific marker expression and Akt activity

**Figure 4:**
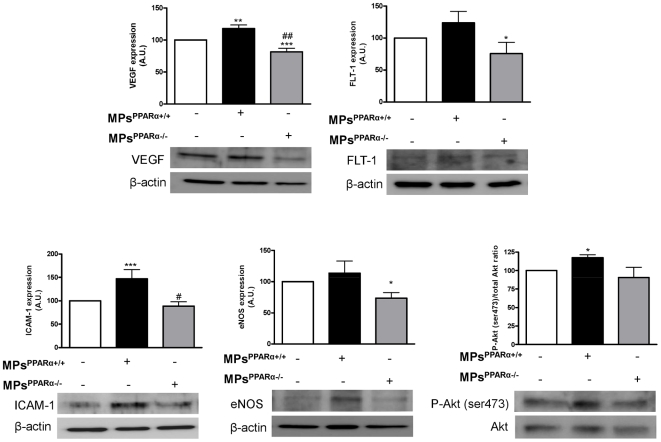
MPs^PPARα+/+^ enhance endothelial-specific marker expression and Akt activation in bone marrow-derived cells. Western blot analysis of EC-markers expression associated with EPC differentiation and Akt phosphorylation. While MPs^PPARα+/+^ enhanced ICAM-1 and VEGF expressions, MPs^PPARα−/−^ significantly attenuated expressions of VEGF, eNOS and FLT-1. Protein expression was normalized by β-actin. Representative Western blots of Akt expression and phosphorylation on its activator site (Ser 473) in EPCs. Histogram showing the increase of P-Akt (ser473)/Akt ratio induced by MPs^PPARα+/+^ in EPCs. *P<0.05, **P<0.01, ***P<0.001 *vs* in the absence of MPs; #*P*<0.05, ##*P*<0.01 *vs* in the presence of MPs^PPARα+/+^. Figures are representative of at least 3 independent experiments for each condition.

We investigated the molecular mechanisms underlying the effects of MPs on EPC differentiation. To further confirm the effects of MPs harboring PPARα on bone marrow-derived cells differentiation at day 7 of growth, we examined the protein expression levels in cellular extracts of pro-angiogenic and EC specific markers and Akt activity in the bone marrow-derived cells (Figure 4). Consistent with the results obtained by q-PCR, Western blot analysis revealed that MPs^PPARα+/+^ significantly enhanced VEGF A but not its receptor FLT-1. Besides, ICAM-1 but not eNOS expression was significantly enhanced upon MPs^PPARα+/+^ stimulation. In contrast, MPs isolated from PPAR^α^-deficient mice significantly decreased the expression of eNOS, VEGF and FLT-1. To demonstrate whether Akt signalling mediates EPC differentiation, bone marrow-derived cells from PPARα WT mice were incubated in the absence or presence of either MPs^PPARα+/+^ or MPs^PPARα−/−^, and Akt phosphorylation on Ser 473 indicative for Akt activity was detected by Western blot analysis. MPs^PPARα+/+^, but not MPs^PPARα−/−^, stimulated Akt phosphorylation indicating an increased activity of Akt in differentiating bone marrow-derived cells (Figure 4).

### MPs^PPARα+/+^ favor *in vitro* capillary-like structure formation of ECs

**Figure 5:**
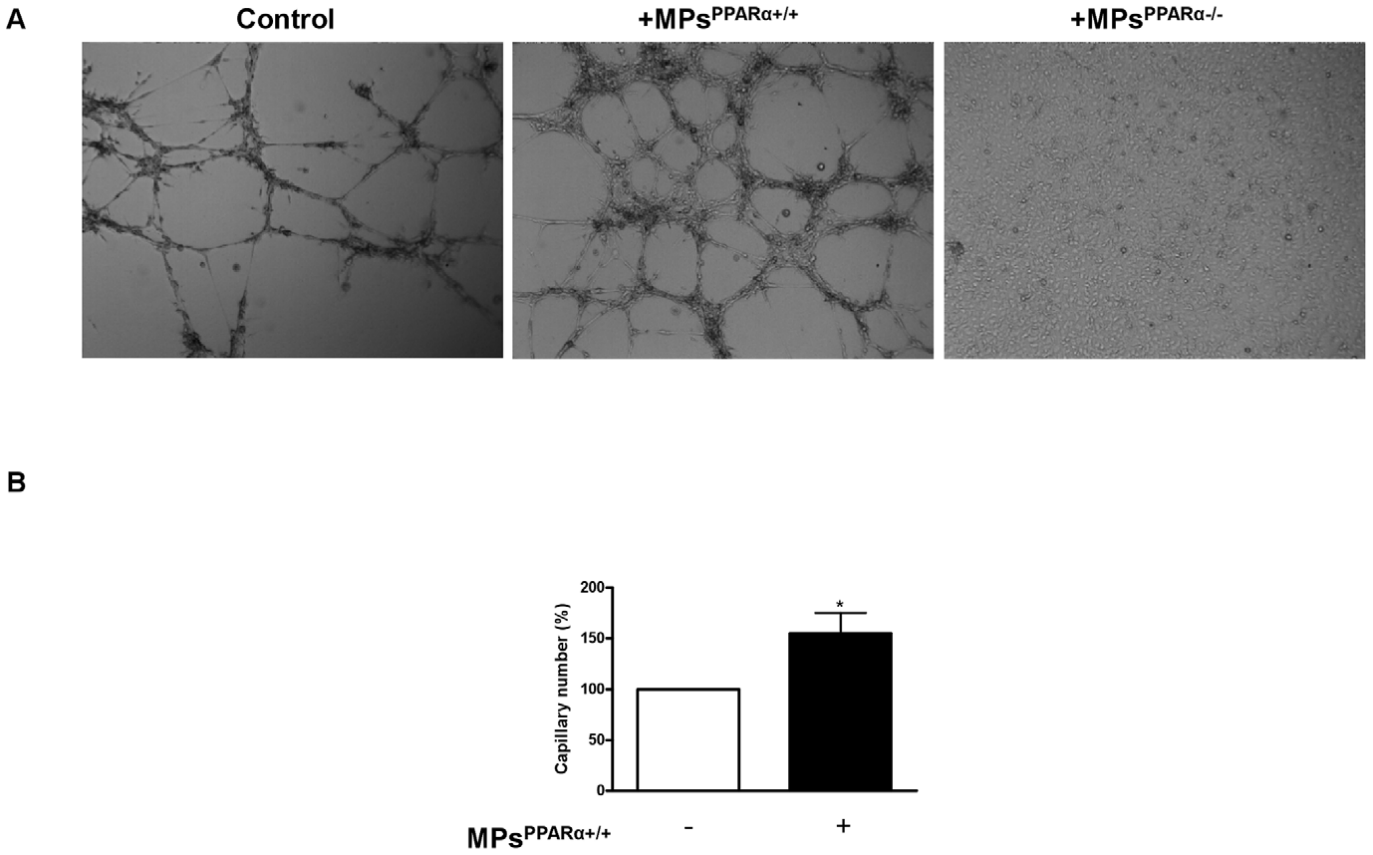
MPs^PPARα+/+^ enhance EC capacity to induce capillary like-structure formation on Matrigel®. (**A**) Phase contrast micrographs of aortic ECs isolated from WT mice. ECs were incubated for 24 hours in the absence or either in the presence of MPs^PPARα+/+^ or MPs^PPARα−/−^. (**B**) Quantification of capillary number revealed that MPs^PPARα+/+^ increased significantly the formation of tubular like-structures of ECs on Matrigel®. Organization of ECs into capillary-like structures on Matrigel® was markedly inhibited when ECs were treated with PPARα deficient MPs. *P<0.05. Figures are representative of at least 3 independent experiments for each condition.

To examine the effects of MPs on the capacity of ECs to form capillary-like structure, ECs were seeded on Matrigel® for 24 hours in the absence or presence of either MPs^PPARα+/+^ or MPs^PPARα−/−^. In the absence of stimulation, ECs from both PPARα WT and KO mice form capillary-like structures suggesting that deletion of PPARα did not affect the angiogenic capacity of ECs (Figure S3A and S3B). Interestingly, MPs^PPARα+/+^ induced significant increases in the number of capillary-like structures (∼55%) relative to untreated ECs from PPARα WT mice (Figure 5A and 5B). To determine whether PPARα accounts for the effects induced by MPs^PPARα+/+^, we assessed the effects of PPARα deletion on MP-induced capillary formation. Capillary formation was absent abolished by MPs^PPARα−/−^ (Figure 5A), suggesting that PPARα harbored by MPs is essential for MP-induced capillary-like structure formation of ECs.

### NF-κB pathway is involved in MP-induced EPC differentiation and *in vitro* EC angiogenesis

NF-κB is a transcription factor that is complexed with I-κBα and β in the cytoplasm, which are selectively phosphorylated, ubiquinated, and degraded, leading to NF-κB translocation. Phosphorylation of I-κBα results in the activation of NF-κB and so allows its translocation to the nucleus.

**Figure 6:**
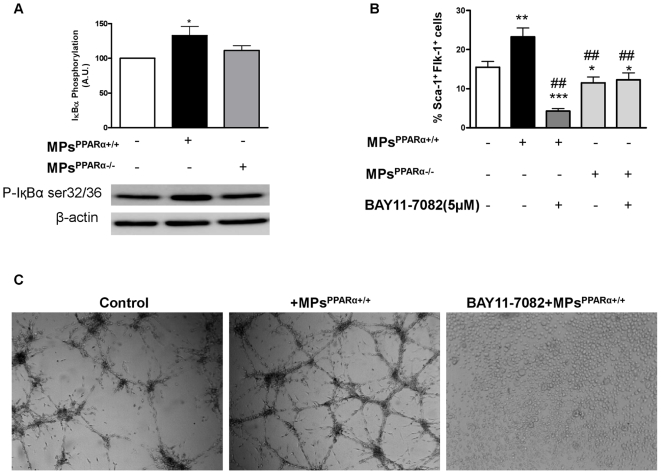
MPs^PPARα+/+^-induced EPC differentiation and *in vitro* angiogenesis of mature ECs involve NF-κB pathway activation. (**A**) Western blot showing that MPs^PPARα+/+^ induced I-κB phosphorylation on activator sites (Ser32/36) in EPCs, indicating that NF-κB pathway is activated. No change in I-κBα phosphorylation was observed in MPs^PPARα−/−^-treated EPCs. *P<0.05 *vs* in the absence of MPs. (**B**) Flow cytometric characterization of EPC differentiation showed that the EPC pretreatment with BAY11-7082 (5 µM) was able to abolish the effect of MPs^PPARα+/+^ on EPC differentiation. However, the decrease in EPC differentiation induced by MPs^PPARα−/−^ was insensitive to BAY11-7082. **P<0.01, ***P<0.001, *P<0.05 *vs* in the absence of MPs; ##*P*<0.01, #*P*<0.05 *vs* in the presence of MPs^PPARα+/+^. (**C**) Formation of MP-induced capillary like-structures of ECs on Matrigel® was markedly inhibited in pretreated ECs with NF-κB pathway inhibitor BAY11-7082 (5 µM). The results indicate the involvement of NF-κB in both MPs^PPARα+/+^-induced EPC differentiation and EC-mediated angiogenesis. Figures are representative of at least 3 independent experiments for each condition.

Western blot analysis of bone marrow-derived cell extracts demonstrated that MPs^PPARα+/+^, but not MPs^PPARα−/−^, significantly enhanced I-κBα phosphorylation on Ser32/36 (Figure 6A), highlighting the activation of NF-κB signaling. To elucidate whether activation of the NF-κB pathway is related to EPC differentiation induced by MPs^PPARα+/+^, we used BAY11-7082 (5 µM), an inhibitor of I-κBα phosphorylation. As shown in Figure 6B, MPs^PPARα+/+^ significantly increase the number of double-positive EPCs for Sca-1^+^/Flk-1^+^ staining. These effects were prevented by incubation with BAY11-7082, suggesting a potential role of the NF-κB pathway in EPC differentiation induced by MPs^PPARα+/+^. In addition, the weak decrease in EPC differentiation induced by MPs^PPARα−/−^ was not modified by BAY11-7082 (Figure 6B) suggesting that NF-κB pathway is not implicated in the effects of MPs^PPARα−/−^.

In another set of experiments, aortic ECs isolated from WT mice were preincubated with BAY11-7082 for 30 minutes, and then later incubated for 24 hours in the absence or presence of MPs^PPARα+/+^. As observed during EPC differentiation experiments, BAY11-7082 also abolished MPs^PPARα+/+^-induced capillary network formation of mature ECs on Matrigel® (Figure 6C), suggesting the involvement of the NF-κB pathway in *in vitro* effects of MPs^PPARα+/+^ on EC-mediated angiogenesis.

### MPs^PPARα+/+^ promote *in vivo* bone marrow-derived cell-mediated angiogenesis

**Figure 7:**
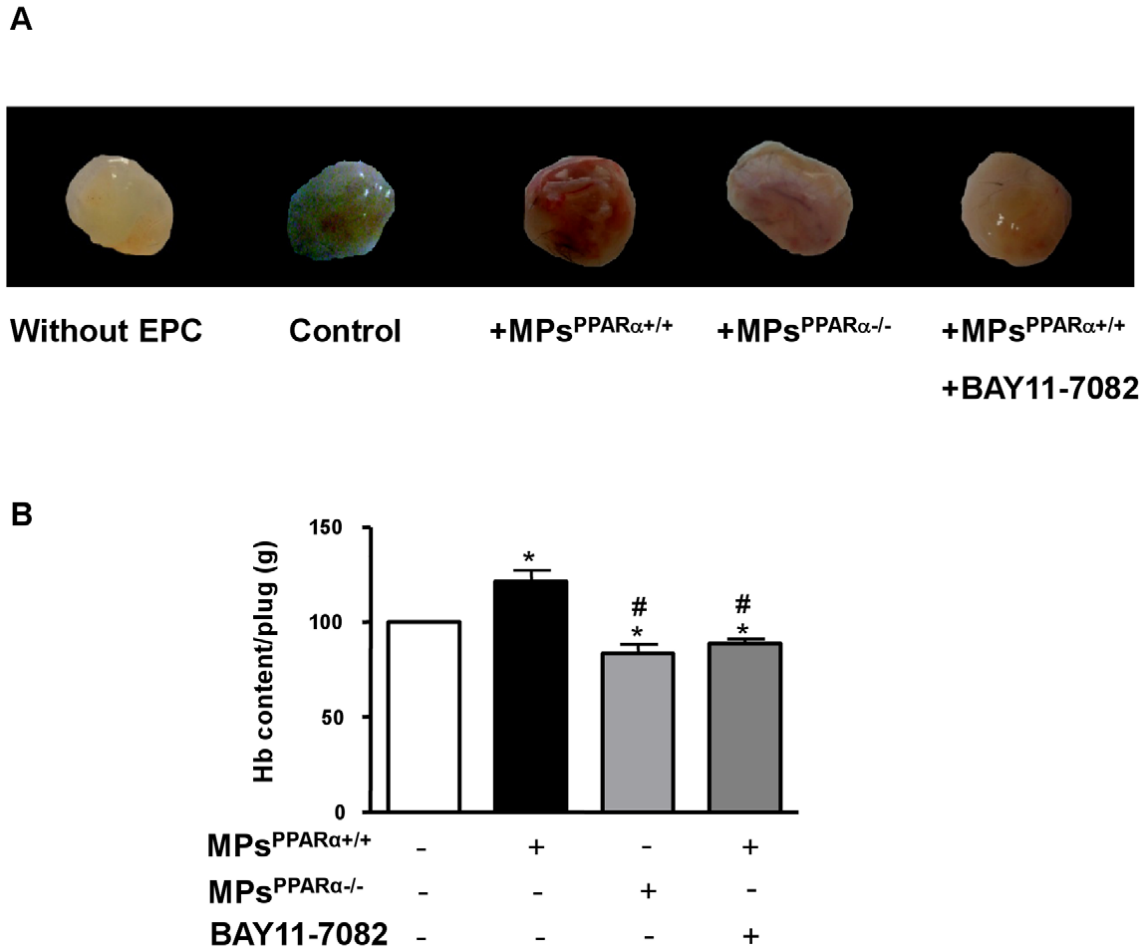
MPs^PPARα+/+^ promote *in vivo* bone marrow-derived cell-mediated angiogenesis. (**A**) MPs^PPARα+/+^-, in the absence or in the presence of BAY11-7082 (5 µM), or MPs^PPARα−/−^-pretreated bone marrow-derived cells were detached, mixed with Matrigel® and b-FGF and injected subcutaneously into WT mice. At day 7, an enhanced neovascularization was observed in plugs containing bone marrow-derived cells pretreated with MPs^PPARα+/+^. (B) Quantitative measurement of hemoglobin was reported as arbitray units/weight of plugs. Hemoglobin content was increased in plugs containing MPs^PPARα+/+^-treated bone marrow-derived cells and decreased in those containing MPs^PPARα+/+^-treated bone marrow-derived cells in the presence of BAY11-7082 or in those containing MPs^PPARα−/−^-treated bone marrow-derived cells. *P<0.05 *vs* in the absence of MPs (CTL). #*P*<0.05 *vs* in presence of MPs^PPARα+/+^. Figures are representative of at least 3 independent experiments for each condition.

Finally, to investigate the effects of MPs on the formation of the new vessels, *in vivo* angiogenesis was studied by using Matrigel® plug assay. At day 7, a blush of vessel proliferation was clearly observed in plugs from mice injected with bone marrow-derived cells treated with MPs^PPARα+/+^, but not with MPs^PPARα−/−^ (Figure 7A). These effects were further quantified by measuring the hemoglobin content of the plugs. Vascularization of the plugs was significantly enhanced (∼25%) in mice injected with bone marrow-derived cells treated with MPs^PPARα+/+^ when compared with those untreated or treated with MPs^PPARα−/−^ (Figure 7B). No further effect was observed at day 15 (not shown). Conversely, when compared with control, hemoglobin content of new-formed blood vessels was significantly reduced (∼20%) in plugs mixed with MPs^PPARα−/−^-treated bone marrow-derived cells. Vascularization of the plugs induced by MPs^PPARα+/+^ was abolished when bone marrow-derived cells were pre-treated with an inhibitor of NF-κB pathway, suggesting a key role of NF-κB activation in the effects evoked by MPs^PPARα+/+^ (Figure 7A and 7B).

## Discussion

Our study demonstrates for the first time that PPARα harbored by MPs mediate the functionality and the differentiation of bone marrow-derived EPCs. The major findings of our study are that MPs^PPARα+/+^: (i) increase EPC differentiation and EC marker expression, but not those of macrophage differentiation; (ii) enhances EPC differentiation that is mediated, at least in part, by PPARα-NF-κB and Akt pathways; (iii) modulate *in vitro* angiogenic-related properties of ECs; and (iv) stimulate *in vivo* bone marrow-derived cell-associated neovascularization. Altogether, these findings provide a novel mechanism underlying EPC differentiation and angiogenesis.

Bone marrow-derived cells contribute to tissue regeneration. In particular, EPCs and macrophages play critical roles in angiogenesis and potentially help to repair injured endothelium [29], [30]. In this study, we show that, after 7 days of culture, bone marrow-derived cells are mainly EPCs (Sca-1^+^CD31^+^) and monocyte/macrophages (Sca-1^+^CD14^+^ and F4/80^+^CD31^+^), but after MPs^PPARα+/+^ treatment, EPC but not macrophage differentiation was observed.

Several studies confirm that MPs favor angiogenesis. Whereas platelet-derived MPs promote angiogenesis through ERK pathway activation [25], endothelial-derived MPs increase capillary-like structure formation via plasminogen generation [23]. We recently reported that MPs from apoptotic/activated lymphocytes evoke angiogenesis through the up-regulation of adhesion and pro-angiogenic factors [26]. However, no data are available concerning circulating MPs. Here, we show that circulating MPs from WT mice favor both *in vitro* and *in vivo* angiogenesis through the modulation of EPC differentiation and angiogenic function, while MPs^PPARα−/−^ decreased both EPC and macrophage differentiations. We cannot rule out the possibility that PPARα deletion can induce other cellular modifications, as previously shown in T cells from PPARα KO mice [31], [32]. Although the mechanisms by which MPs^PPARα+/+^ induces both EPC differentiation and neovascularization are not completely elucidated, we demonstrated that effects evoked by MPs^PPARα+/+^ involve NF-κB signaling that was associated with Akt activation. Moreover, PPARα is not transferred from MPs into bone marrow-derived cells since PPARα expression is not enhanced in these cells after treatment with MPs^PPARα+/+^. On the other hand, PPARα is expressed and functional in bone marrow-derived cells, suggesting that lipids harbored by MPs^PPARα+/+^, but not those carried by MPs^PPARα−/−^, may act as endogenous ligands. In agreement with previous studies [33], [34], it is possible that mRNA or miRNA delivery from MPs^PPARα+/+^ to target cells (in this case bone marrow-derived cells or ECs) may be responsible for changes of their phenotype. In this context, it has been described that MPs from EPCs may activate angiogenic program in ECs [34]. Further experiments are necessary to determine the molecular mechanism by which MPs transfer their message.

Activated PPARα is required during *in vitro* differentiation of murine stem cells towards cardiac cells [16], [17] while PPARα activators induce keratinocyte differentiation [35] and phenotypic modulation of rat oval cells toward the hepatocyte lineage [36]. Biscetti and coworkers [19] recently reported that iloprost-induced angiogenesis and VEGF up-regulation are dependent on the presence and proper function of PPARα gene. Besides, PPARγ agonists favorably modulated bone marrow-derived progenitor cells to promote endothelial lineage [37]. Our findings demonstrate that the PPARα gene is necessary for MPs to develop pro-angiogenic activity since circulating MPs form PPARα deficient mice did not induce either EPC differentiation, *in vivo* neovascularization or *in vitro* EC angiogenesis. In addition, the selective pharmacological PPARα agonist WY-14643 evoked EPC differentiation in bone marrow-derived cells from WT PPARα mice, but not in those derived from KO PPARα mice, suggesting that PPARα activation accounts for pro-angiogenic effects on EPCs.

To elucidate the potential molecular mechanisms underlying EPC differentiation and pro-angiogenic properties of MPs harboring PPARα, we evaluated the expression of pro-angiogenic factors at the mRNA level in *ex vivo* expanded bone marrow-derived cells pretreated with MPs^PPARα+/+^ or MPs^PPARα−/−^. A significant increase of mRNA levels of pro-angiogenic factors, such as VEGF A, Ang-2 and SDF-1 was observed in bone marrow-derived cells treated with MPs^PPARα+/+^, suggesting that MPs from PPARα mice may be involved in the enhanced homing ability of EPCs and in their pro-angiogenic abilities. Since SDF-1 pretreatment during EPC expansion stimulates EPC adhesion and increases the efficiency of cell therapy for ischemic vascular diseases, increases EPC number and recruitment of bone marrow-derived EPCs into site of ischemia [38], one can advance the hypothesis that MPs^PPARα+/+^ can be used as an expansion agent of EPCs. In addition, expressions of EC markers such as VE-cadherin, PECAM-1, and ICAM-1 were increased, indicating that MPs^PPARα+/+^ evoke differentiation of bone marrow-derived cells towards an endothelial lineage. In contrast, TSP-1 which possesses potent anti-angiogenic activity [39] was down-regulated in bone marrow-derived cells treated by MPs^PPARα+/+^ and conversely up-regulated in pretreated bone marrow-derived cells with MPs^PPARα−/−^. These results emphasize the key role of PPARα in modulating angiogenic potential of bone marrow-derived cells.

Supporting the results obtained with bone marrow-derived cells, MPs^PPARα+/+^ also significantly enhanced the expression of the pro-angiogenic factor VEGF and EC marker ICAM-1, whereas eNOS, VEGF and FLT-1 protein levels were significantly decreased in bone marrow-derived cells treated with MPs^PPARα−/−^. These data are in accordance with those recently reported by Biscetti et al [18] showing that PPARα activation evokes angiogenesis through VEGF up-regulation. Collectively, these results indicate that MPs^PPARα+/+^ are able to promote the angiogenic program of both EPCs and ECs so as to enhance their proangiogenic properties. However, it remains to be determined whether MPs^PPARα+/+^ act directly on bone marrow-derived cells to induce EPC differentiation and pro-angiogenic factor release (i.e. VEGF) or whether bone marrow-derived cells play a paracrine angiogenic role. In this respect, it has been shown the essential paracrine role of recruited bone marrow-derived circulating cells occurs by secreting pro-angiogenic factors rather than serving as endothelial progenitors in an adult model of neovascularization [29]. These authors have shown that SDF-1 secreted by perivascular cells in response to VEGF is responsible for positioning of bone marrow-derived circulating cells near to blood vessels. In this study, we show that MPs^PPARα+/+^ enhances both VEGF and SDF-1 mRNA, suggesting that similar mechanisms may occur.

The anti-inflammatory actions of PPARs have been largely described to inhibition of the NF-κB pathway [40], [41]. Surprisingly, in our study the effects of MPs^PPARα+/+^ on EPC differentiation and EC angiogenesis are sensitive to NF-κB inhibition. Furthermore, MPs^PPARα+/+^ effects were associated with increased phosphorylation of I-κBα (Ser32/36) in bone marrow-derived cells. These findings suggest a positive regulation of NF-κB pathway by PPARα, as described in other studies [42]. In this context, PPARs have been recently reported to be involved in neural stem cells (NSC) acquisition of a specific fate, and the control of NSC proliferation, migration and differentiation through NF-κB pathway [43]. A similar signaling through PPARα significantly may contribute to EPC differentiation via stimulation of NF-κB pathway.

The Akt pathway plays a central role in the functioning of mature ECs. Thus, activation of Akt promotes endothelial cell survival by inhibiting apoptosis and is involved in EPC differentiation [44]. Our study not only extends these findings by demonstrating that Akt activation is associated with EPC differentiation, but also suggests Akt as a target for MPs^PPARα+/+^ in modulating EPC differentiation and functions. On the basis of these findings, it is possible that one of the mechanisms that mediates EPC differentiation induced by MPs^PPARα+/+^ is the crosstalk between PPARα-NF-κB and Akt pathways.

Finally, to further validate our obtained data concerning the effects of MPs^PPARα+/+^ on *in vitro* EPC differentiation, we investigated the *in vivo* effects of untreated or MP-treated bone marrow-derived cells on neovascularization. MPs^PPARα+/+^-treated bone marrow-derived cells enhanced the formation of new vessels as assessed by increased hemoglobin content in Matrigel® plugs; these effects were attenuated by inhibition of the NF-κB pathway. These findings demonstrate that isolated bone marrow-derived cells expanded by *in vitro* MP treatment are able to generate *in vivo* functional vessels and confirm our results obtained *in vitro.*

In conclusion, the results of this study confirm the pro-angiogenic ability of circulating MPs harboring PPARα, which are able to induce *in vitro* EPC differentiation and angiogenesis through the NF-κB pathway. Therefore, MP treatment may significantly contribute to EPC differentiation and stimulation of neovascularization following ischemic injury. The PPARα-NF-κB-Akt pathways may play a pivotal stimulatory role in neovascularization, which is, at least in part, mediated through bone marrow-derived EPCs. Improvement of EPC differentiation may greatly aid reparative neovascularization.

## Materials and Methods

### Circulating MP isolation

All animal studies were carried out using approved institutional protocols and were conformed the *Guide for the Care and Use of Laboratory Animals* published by US National Institutes of Health (NIH Publication No. 85-23, revised 1996). Mice (6 to 8 week old) were anesthetized by isofluorane for all procedures. Circulating MPs were isolated from venous citrated blood collected from PPARα null (KO) and corresponding wild*-*type (WT). PPARα KO mice were generated by the group of Gonzalez, as previously described [45]. Briefly, blood was centrifuged at 1,900 *g* for 3 minutes and platelet-rich plasma was separated from whole blood. Then, platelet-rich plasma was centrifuged at 5,000 *g* for 4 minutes to obtain platelet-free plasma (PFP). Sixty µL of PFP were frozen and stored at −80°C until subsequent use. In order to pellet MPs for *in vitro* and *in vivo* studies, circulating MPs were concentrated from PFP by three series of centrifugations at 21,000 *g* for 45 minutes and resuspended in 0.9% saline salt solution and stored at 4°C until subsequent use. The physiological circulating concentration of mice MPs was used in all experiments. Washing medium for the last supernatant was used as control.

### Characterization of MP phenotype

Membrane MP subpopulations were discriminated in PFP according the expression of membrane-specific antigens. Phenotype of endothelial MPs was performed using anti-CD54 labeling, characterization of platelet, leukocyte and erythrocyte MPs was performed using anti-CD61, anti-CD45, anti-Ter-119/erythroid cell and anti-CD133 labeling, respectively. Irrelevant mouse IgG was used as an isotype-matched negative control for each sample.

For numeration studies, 8 µL of PFP were incubated with either 1 µL of specific antibody (BioLegend, San Diego, CA). After 45 minutes of incubation at room temperature, samples were diluted in 300 µL of 0.9% saline salt solution. Annexin V (BioVision Inc., Mountain View, CA) binding was used to numerate phosphatidylserine-expressing circulating MPs (2 µL of annexin V/5 µL PFP). Then, in order to enumerate MPs, an equal volume of sample and Flowcount beads were added and samples were analyzed in a flow cytometer 500 MPL system (Beckman Coulter, Roissy, France) as previously described [46], [47].

### Isolation, culture and characterization of EPCs

Bone marrow-derived cells were obtained by isolating mononuclear cells from bone marrow of WT or KO mice (6 to 8 week old) using Histopaque® 1077 (Sigma-Aldrich, St Louis, MO) density-gradient centrifugation. Briefly, immediately after isolation, total mononuclear cells (10^6^ cells/cm^2^) were plated on culture dishes coated with (10 µg/mL) fibronectin (BD Biosciences, San Jose, CA) and maintained in EGM-2 endothelial medium BulletKit system (Lonza, Walkersville, MD) consisting of endothelial basal medium supplemented with 5% FBS, VEGF, FGF-2, EGF, IGF-1, ascorbic acid, gentamicin sulfate amphotericin, hydrocortisone and heparin. After 24 h, non-adherent cells were removed by washing with PBS, and adherent cells were cultured in EGM-2 endothelial medium in the presence of either washing medium, MPs from WT (MPs^PPARα+/+^) or KO (MPs^PPARα−/−^) mice, for the whole culture period (7 days). Cells were treated at circulating levels of MPs detected in the blood of mice as previously performed [46], [47]. In another set of experiments, the specific agonist PPARα, WY-14643 (Calbiochem, London, UK; 5 µM) was used to activate PPARα in bone marrow-derived cells either from WT or KO mice. To analyze cell phenotype, extensive characterization of isolated cells at day 0 and after 7 days of culture was performed. Labelled cells were analyzed by flow cytometry using the following Abs: anti-CD133-FITC (eBioscience, San Diego, CA), anti-CD14-FITC (BioLegend, San Diego, CA), anti-Ly-6A/E (Sca-1)-PE/Cy7, anti-Flk-1 (VEGFR2)-PE, or anti-F4/80-PE/Cy7, anti-CD45-PE/Cy5 (BioLegend), anti-CD31 (BD Biosciences, San José, CA) followed by incubation with goat anti-rat IgG-FITC (Southern Biotech, Birmingham, AL).

After 7 days of treatment with MPs, adherent EPCs were characterized by confocal microscopy and by flow cytometry. Briefly, adherent cells were incubated with 2.5 µg/mL 1,1′-dioctadecyl-3,3,3′,3′-tetramethyl-indocarbocyanine-labeled acetylated-LDL (DiI-Ac-LDL) (Harbor Bioproducts, Norwood, MA) for 3 hours and then, fixed with 2% paraformaldehyde and counterstained with 10 µg/mL FITC-labeled lectin from *Bandeiraea simplicifolia* (Sigma-Aldrich) for 1 hour at 37°C. Nuclei were counterstained with 4′,6-diamidino-2-phenylindole (DAPI) (Santa Cruz Biotechnology, Santa Cruz, CA) at room temperature for 5 min. Double positive cells (DiI-ac-LDL^+^/FITC-lectin^+^), identified as EPCs by confocal microscopy and by three independent investigators, were counted in 5 randomly selected high-magnification fields of each culture slide by using a computer-based program.

In parallel, after 7 days of treatment, adherent cells were detached with scratching with accutase (Sigma-Aldrich), and then incubated for 45 minutes at room temperature simultaneously with anti-Ly-6A/E (Sca-1)-PE/Cy7 and anti-Flk-1-PE Abs or anti-F4/80-PE/Cy7 and anti-CD31 followed incubation with goat anti-rat IgG-FITC. Isotype-matched antibodies served as controls. After washing, cells were analyzed by flow cytometer 500 MPL system (Beckman Coulter). Ten thousand events were acquired by sample.

### Aortic EC isolation and characterization

Aortic ECs were isolated from either PPARα+/+ or PPARα−/− mice (6 to 8 week old) as previously described [48]. The endothelial phenotype of primary aortic ECs was verified by flow cytometry (data not shown).

### In vitro capillary network formation on Matrigel®

ECs isolated from either PPARα+/+ or PPARα−/− aorta were seeded (10^5^ cells/cm^2^) on culture dishes coated with extracellular matrix gel from Engelbreth-Holm-Swarm murine sarcoma (Matrigel®, Sigma-Aldrich). Briefly, 200 µL of Matrigel® substrate diluted with serum-free medium (1∶1 dilution) was added into a four-well plate and allowed to solidify for 1 hour at 37°C. Then, cells were incubated with medium containing 5% of FBS and allowed to adhere for 1 hour. ECs were incubated for 24 hour in the absence or in the presence of MPs from either PPARα+/+ or PPARα−/− mice. Capillary-like structures formation was examined using an inverted phase contrast microscope (MOTIC AE21) and quantified using ImageJ software.

### In vivo Matrigel® plug assay

For *in vivo* studies of bone marrow-derived cell-induced angiogenesis, isolated bone marrow-derived cells from WT mice were incubated in the absence or in the presence of MPs isolated from PPARα+/+ or PPARα−/− mice for 7 or 15 days. Matrigel® plugs were prepared on ice by mixing 500 µL of Matrigel® (Sigma-Aldrich) with recombinant bFGF (300 ng/mL, Peprotech, Rocky Hill, NJ) and bone marrow-derived cells treated or untreated with MPs. This mixture was injected subcutaneously on the back of WT mice. At day 7, Matrigel® plugs were removed and homogenized in lysis buffer and incubated for 24 hour at 4°C and then, disrupted with a Polytron (PRO250, Monroe, CT). Hemoglobin concentration was measured in the supernatants with Drabkin's reagent (Sigma-Aldrich) according to the manufacturer's instructions.

### Quantitative-PCR (q-PCR) analysis

Bone marrow-derived cells isolated from PPARα+/+ mice were grown for 7 days in the absence or presence of MPs isolated from PPARα+/+ or PPARα−/− mice. Cells were washed with PBS and lysed with Tri-reagent lysis buffer. Cell lysates were frozen in liquid N_2_ and used to investigate the expression of mRNA for 52 transcripts related to differentiation and angiogenesis by quantitative real-time reverse transcription-polymerase chain reaction (q-PCR) (Table S1). q-PCR analyses were carried out by Service Commun de Cytométrie et d'Analyses Nucléotidiques from Angers University, using a Chromo 4™ (Bio-Rad, Hercules, CA) and SYBR Green detection. Primers were designed using Primer3 software (http://frodo.wi.mit.edu/cgi-bin/primer3/primer3_www.cgi). Quantifications were realized according to the ΔCt method and the relative gene expression levels were normalized using the geometric mean of three housekeeping genes as previously described [49].

### Western blot

Adherent cells were homogenized and lysed. Proteins (20 µg) were separated on 10% SDS-PAGE. Blots were probed with anti-endothelial NOS (eNOS), (BD Biosciences), ICAM-1, VEGF and fms-like tyrosine kinase (FLT-1) (Santa Cruz Biotechnology), phosphorylated (Ser32/36)-I-κBα, (US Biological, Swampscott, MA), Akt, Phospho-Akt (ser473), (Cell signaling, Beverly, MA) Abs. A monoclonal mouse anti-β-actin Ab (Sigma-Aldrich) was used at 1/2,000 dilution to visualize protein gel loading. The membranes were then washed at least three times in Tris buffer solution containing 0.05% Tween and incubated for 1 hour at room temperature with the appropriate horseradish peroxidase (HRP)-conjugated secondary antibody (Amersham Biosciences, Piscataway, NJ). The protein-antibody complexes were detected by ECL plus (Amersham) according to the protocol of the manufacturer.

### Data analysis

Data are represented as mean ± SEM; *n* represents the number of mice. Statistical analyses were performed by Mann-Whitney U-tests (non-parametric) using Prism software package 4.00 (GraphPAD Software, San Diego, CA). *P*<0.05 was considered to be statistically significant.

## Supporting Information

Figure S1 Flow cytometry characterization of isolated cells derived from bone marrow from PPARα+/+ mice at day 0 and after 7 days of culture. At day 7, cells displaying a double labeling for Sca-1/CD31, Sca-1/CD45, Sca-1/CD14 and F4/80/CD31 were significantly increased when compared to cells at day 0. Also, Sca-1/Flk-1 and CD133/Flk-1 cells were slightly increased when compared to cells at day 0. *P<0.05, **P<0.01, ***P<0.001 vs isolated cells at day 0. Figure is representative of at least 6 independent experiments for each condition.
(0.12 MB TIF)

Figure S2 PPARα activation increased EPC differentiation. (A) Western blot showing that, after 7 days of culture, EPCs undergoing differentiation express PPARα. MPs treatment of EPCs had no effect on PPARα expression compared with control indicating that of PPARα transfer did not take place. Protein expression was normalized by β-actin. (B) Flow cytometric analysis of EPC differentiation showed that PPARα activation (treatment of EPCs isolated from WT mice with WY-14643 (5 µM), a specific PPARα agonist) increased significantly Sca-1+/Flk-1+ cells indicating an increased differentiation. (C) Treatment of EPCs isolated from PPARα−/− with PPARα agonist had no effect on EPC differentiation in vitro. (D) Sca-1+/Flk-1+ percentage of cells isolated from PPARα−/− was not modified in the presence of MPsPPARα+/+. *P<0.05 vs untreated cells. Figures are representative of at least 3 independent experiments for each condition.
(0.18 MB TIF)

Figure S3 Organization of ECs into capillary-like structures on Matrigel® was not impaired in ECs isolated from PPARα−/−mice. (A) Phase contrast micrographs of aortic ECs isolated from PPARα−/− and their corresponding WT mice. ECs were grown for 24 hours on Matrigel matrix. (B) Quantification of capillary number revealed that there was no significative difference in the number of formed capillary-like structures on Matrigel® between PPARα−/− and their corresponding WT mice. Figures are representative of at least 4 independent experiments for each condition.
(0.71 MB TIF)

Table S1 Genes analyzed by qRT-PCR.
(0.03 MB DOC)
