# Supplementary figures and images for: PPARα Is Essential for Microparticle-Induced Differentiation of Mouse Bone Marrow-Derived Endothelial Progenitor Cells and Angiogenesis
Source: PLoS One. 2010 Aug 25;5(8):e12392. doi: 10.1371/journal.pone.0012392 (PMC2928272; doi:10.1371/journal.pone.0012392)

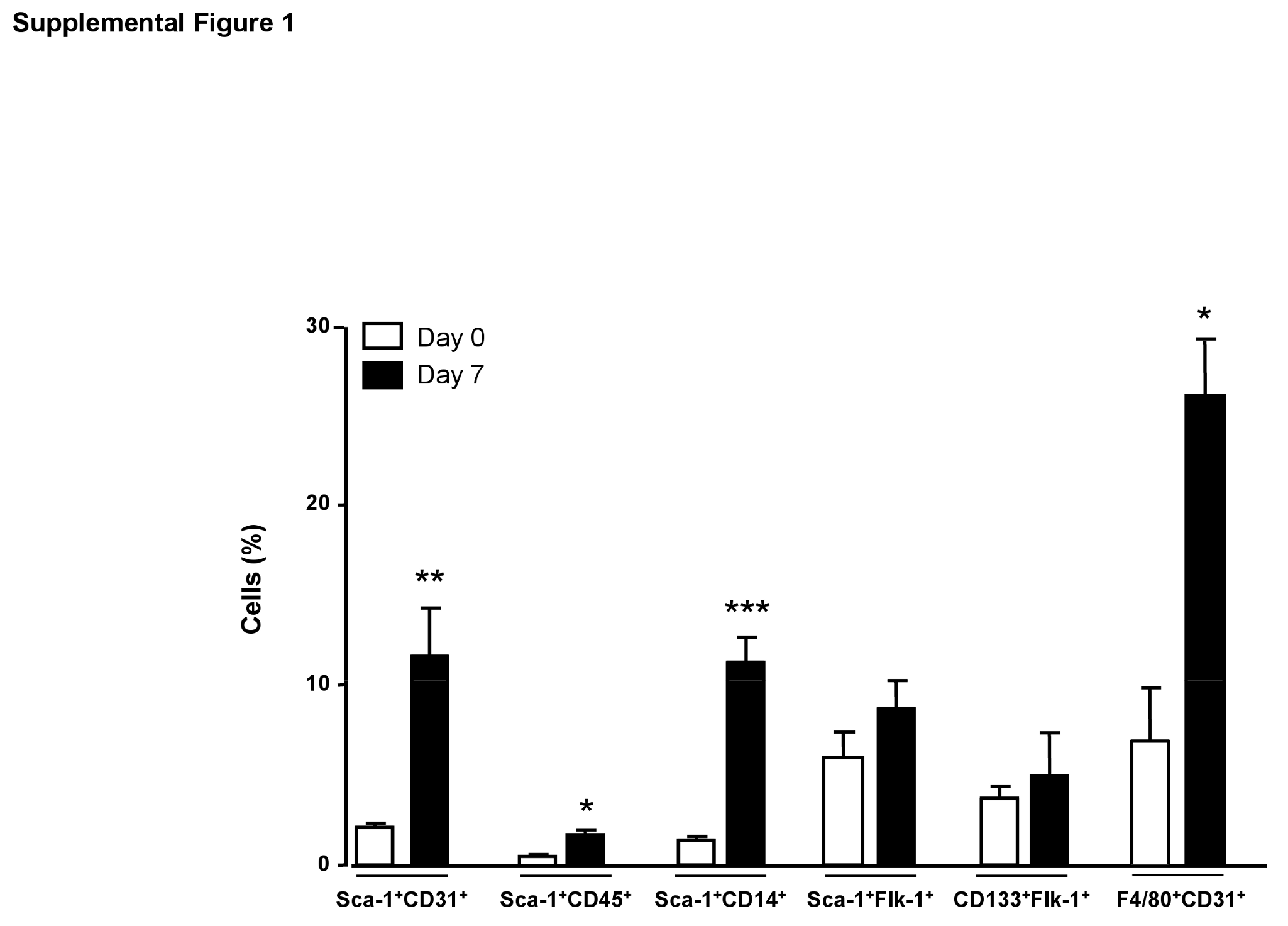

Supplement: Figure S1 — Flow cytometry characterization of isolated cells derived from bone marrow from PPARα+/+ mice at day 0 and after 7 days of culture. At day 7, cells displaying a double labeling for Sca-1/CD31, Sca-1/CD45, Sca-1/CD14 and F4/80/CD31 were significantly increased when compared to cells at day 0. Also, Sca-1/Flk-1 and CD133/Flk-1 cells were slightly increased when compared to cells at day 0. *P<0.05, **P<0.01, ***P<0.001 vs isolated cells at day 0. Figure is representative of at least 6 independent experiments for each condition. (0.12 MB TIF) [file pone.0012392.s001.tif]

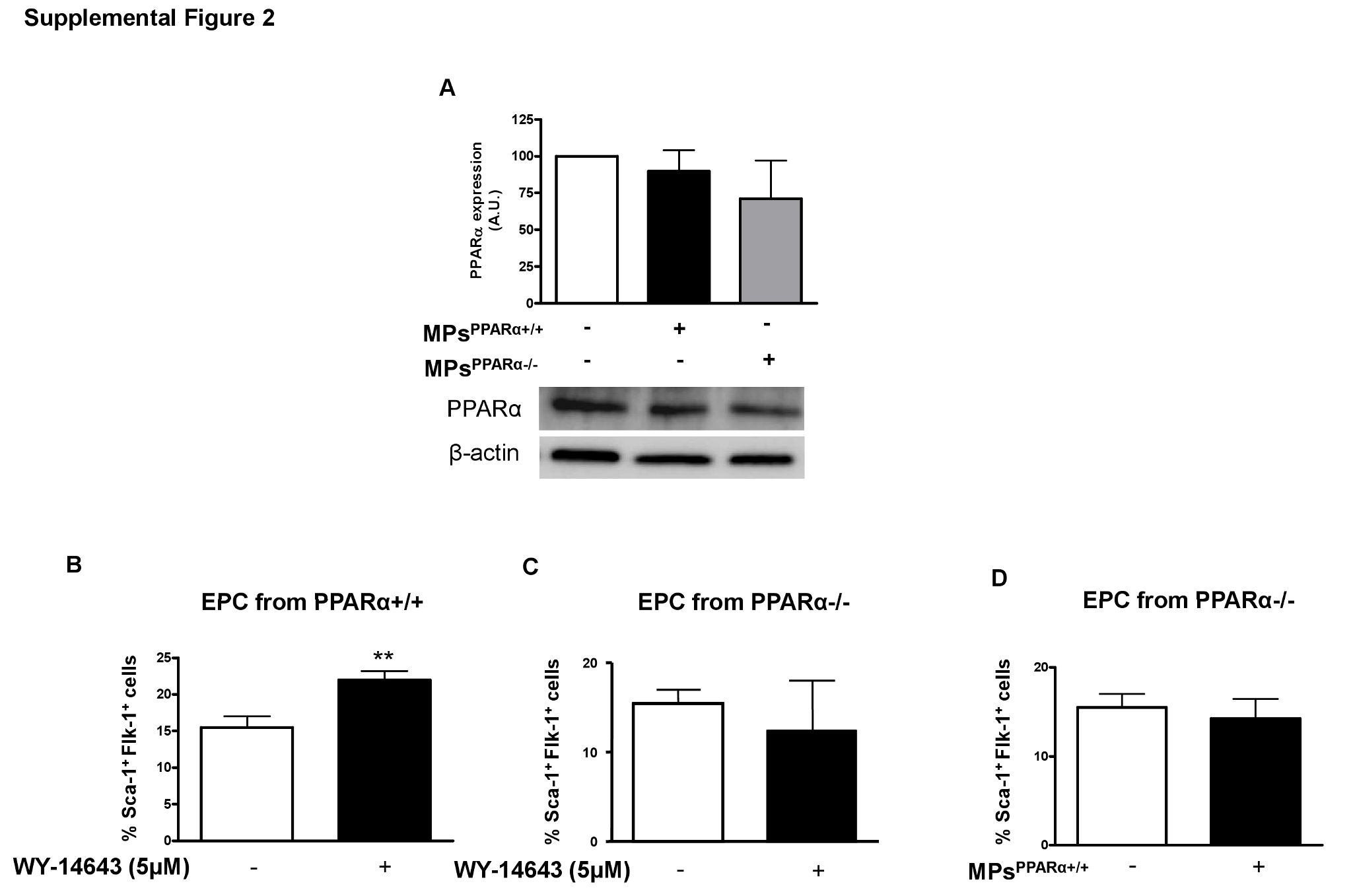

Supplement: Figure S2 — PPARα activation increased EPC differentiation. (A) Western blot showing that, after 7 days of culture, EPCs undergoing differentiation express PPARα. MPs treatment of EPCs had no effect on PPARα expression compared with control indicating that of PPARα transfer did not take place. Protein expression was normalized by β-actin. (B) Flow cytometric analysis of EPC differentiation showed that PPARα activation (treatment of EPCs isolated from WT mice with WY-14643 (5 µM), a specific PPARα agonist) increased significantly Sca-1+/Flk-1+ cells indicating an increased differentiation. (C) Treatment of EPCs isolated from PPARα−/− with PPARα agonist had no effect on EPC differentiation in vitro. (D) Sca-1+/Flk-1+ percentage of cells isolated from PPARα−/− was not modified in the presence of MPsPPARα+/+. *P<0.05 vs untreated cells. Figures are representative of at least 3 independent experiments for each condition. (0.18 MB TIF) [file pone.0012392.s002.tif]

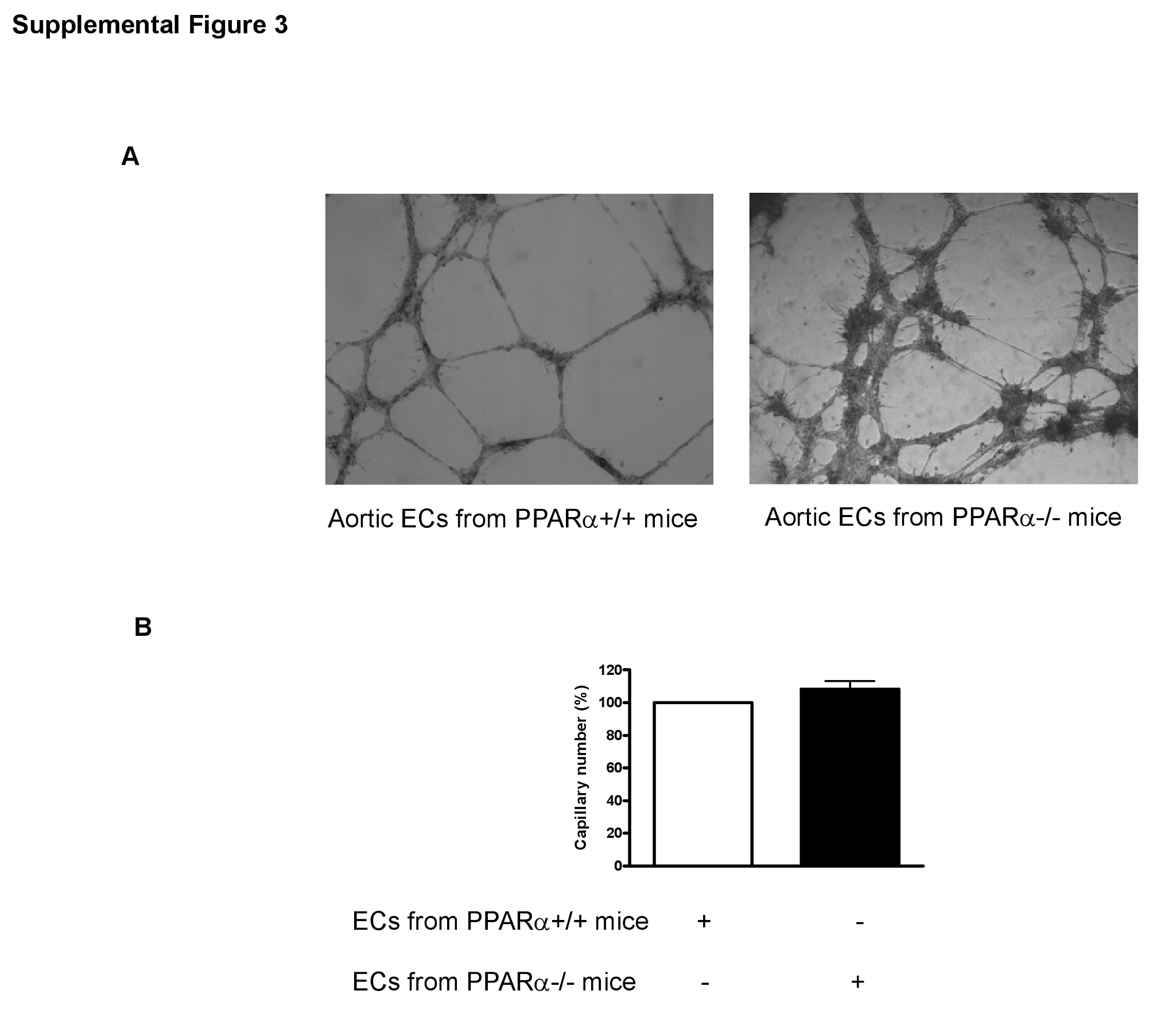

Supplement: Figure S3 — Organization of ECs into capillary-like structures on Matrigel® was not impaired in ECs isolated from PPARα−/−mice. (A) Phase contrast micrographs of aortic ECs isolated from PPARα−/− and their corresponding WT mice. ECs were grown for 24 hours on Matrigel matrix. (B) Quantification of capillary number revealed that there was no significative difference in the number of formed capillary-like structures on Matrigel® between PPARα−/− and their corresponding WT mice. Figures are representative of at least 4 independent experiments for each condition. (0.71 MB TIF) [file pone.0012392.s003.tif]
