# Supplementary material for: PPARα Is Essential for Microparticle-Induced Differentiation of Mouse Bone Marrow-Derived Endothelial Progenitor Cells and Angiogenesis
Source: PLoS One. 2010 Aug 25;5(8):e12392. doi: 10.1371/journal.pone.0012392 (PMC2928272; doi:10.1371/journal.pone.0012392)
Supplement: Table S1 — Genes analyzed by qRT-PCR. (0.03 MB DOC) [file pone.0012392.s004.doc]

| Alk-1 | eNOS | FLT1 | PDGFB |
| --- | --- | --- | --- |
| α5 integrin | ephrine b2 | GROa | PECAM-1 |
| ANG-1 | EPO | GROg | PEDF |
| ANG-2 | E-selectin | HGF | TGFb1 |
| Angiogenin | FGF1 | ICAM-1 | TGFb2 |
| β3 integrin | FGF10 | IGF-1 | TNFα |
| CCL2 (MCP-1) | FGF2 | IL-1β | TSP1 |
| CCL5 (RANTES) | FGF3 | IL-6 | VCAM-1 |
| CCR2 | FGF5 | IL8RB | VE-cadherin |
| CD148 | FGF7 | KDR | VEGF A |
| CXCL12 (SDF-1) | FGF8 | MMP2 | VEGF B |
| CXCL5 (ENA-78) | FGFR1 | MT1-MMP1 | VEGF C |
| Edg-1 | FGFR2 | PDGFA | VEGF D |
